# Polymer-Based Scaffolds as an Implantable Material in Regenerative Dentistry: A Review

**DOI:** 10.3390/jfb16030080

**Published:** 2025-02-24

**Authors:** Lubos Lesko, Petra Jungova, Martina Culenova, Andrej Thurzo, Lubos Danisovic

**Affiliations:** 1 Institute of Medical Biology, Genetics and Clinical Genetics, Faculty of Medicine, Comenius University in Bratislava, Sasinkova 4, 811 08 Bratislava, Slovakia; lesko13@uniba.sk (L.L.); martina.culenova@fmed.uniba.sk (M.C.); 2 Department of Orthodontics, Regenerative and Forensic Dentistry, Faculty of Medicine, Comenius University in Bratislava, Dvořákovo nábrežie 4, 811 02 Bratislava, Slovakia; jungova2@uniba.sk (P.J.); andrej.thurzo@fmed.uniba.sk (A.T.); 3 National Institute of Rheumatic Diseases, Nábrežie I. Krasku 4, 921 12 Piešťany, Slovakia; 4 Regenmed Ltd., Medena 29, 811 01 Bratislava, Slovakia

**Keywords:** regenerative dentistry, tissue engineering, polymer-based scaffolds, dental tissue regeneration

## Abstract

Polymer-based scaffolds have emerged as transformative materials in regenerative dentistry, enabling the restoration and replacement of dental tissues through tissue engineering approaches. These scaffolds, derived from natural and synthetic polymers, mimic the extracellular matrix to promote cellular attachment, proliferation, and differentiation. Natural polymers such as collagen, chitosan, and alginate offer biocompatibility and bioactivity, while synthetic alternatives like polylactic acid (PLA) and polycaprolactone (PCL) provide tunable mechanical properties and degradation rates. Recent advancements highlight the integration of bioactive molecules and nanotechnology to enhance the regenerative potential of these materials. Furthermore, developing hybrid scaffolds combining natural and synthetic polymers addresses biocompatibility and mechanical strength challenges, paving the way for patient-specific treatments. Innovations in 3D bioprinting and stimuli-responsive biomaterials are expected to refine scaffold design further, improving therapeutic precision and clinical outcomes. This review underscores the critical role of polymer-based scaffolds in advancing regenerative dentistry, focusing on their applications, advantages, and limitations.

## 1. Introduction

The oral cavity represents a complex anatomical structure where physical, mechanical, and biochemical forces heavily influence its inner components [1]. Dental tissues are significant parts of oral cavities and their proper functioning has a direct effect on human wellbeing. Firstly, they take part in food intake and further processing of nourishment into other parts of the digestive system. Secondly, as the oral cavity is an entrance for many outer pathogens and itself is heavily colonized by bacteria, healthy and functioning dental tissues also help to maintain a balanced state in the mouth [2]. Dental tissues are also significant for facial esthetics. Acute trauma, infections, and congenital malformations are only a few examples that lead to damage of dental tissues [3,4].

**Figure 1:**
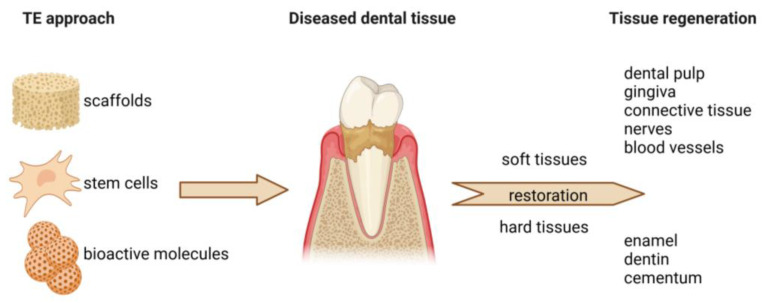
The principles of regenerative dentistry. Regenerative dentistry employs the application of various biomaterials in the form of scaffolds. Together with stem cells and bioactive molecules (e.g., growth factors, hormones), cell-seeded scaffolds can be used to repair and enhance the regeneration of both soft and hard dental tissues.

Regenerative dentistry is an emerging field focused on restoring dental tissues through biological mechanisms, including tissue engineering, stem cell therapy, and biomaterial applications (Figure 1). Unlike traditional dentistry, which often relies on mechanical and synthetic solutions, regenerative dentistry stimulates the body’s natural healing processes to repair or replace damaged dental tissues such as enamel, dentin, pulp, cementum, periodontal ligament, and alveolar bone [5].

The scope of regenerative dentistry encompasses virtually all areas of dentistry and its various components, with a distinct approach to the therapy of damaged or missing parts. This field includes dental pulp regeneration, which involves techniques aimed at regenerating dental pulp tissue in cases of pulpitis or trauma. Additionally, periodontal regeneration focuses on strategies to restore periodontal tissues lost due to periodontal disease, including the regeneration of the periodontal ligament, cementum, and alveolar bone [5,6].

Craniofacial reconstruction is another critical aspect, where engineering craniofacial structures, such as the mandible and maxilla, are achieved using stem cells and scaffolds for patients with congenital anomalies or trauma. Furthermore, bone regeneration techniques are employed to regenerate bone tissue in the jaw and surrounding areas, addressing issues like bone loss resulting from periodontal disease or tooth extraction [7].

Lastly, soft tissue engineering plays a vital role in this field. It focuses on regenerating soft tissues, such as gingiva, through stem cells and biomaterials. This approach aims to enhance esthetic and functional outcomes in dental procedures, highlighting the comprehensive nature of regenerative dentistry in improving patient care and outcomes [8].

The field of tissue engineering began to develop in the late 20th century. Its primary goal was to improve bone regeneration by combining various elements such as scaffolds, stem cells, and growth factors [9]. Scaffolds serve as temporary structures that support the growth of cells and tissues. Stem cells can differentiate into various cell types, including osteoblasts, which are responsible for bone formation. Growth factors are biologically active molecules that stimulate the growth and regeneration of tissues. This approach aims to create a biocompatible environment that mimics the body’s natural conditions and supports healing and bone growth. In this way, tissue engineering seeks to provide effective solutions for treating bone defects and injuries that cannot heal independently [6,10].

Biomaterials play a crucial role in regenerative dentistry. They can be categorized into natural and synthetic polymers. While synthetic polymers are artificially produced under laboratory conditions, natural polymers widely occur in nature. In addition, they can be extracted from both plants and animals. Each type has advantages and limitations concerning biocompatibility, degradation rate, and mechanical properties [11,12,13].

This article explores the development, advantages, and challenges of using natural and synthetic polymer-based scaffolds in regenerative dentistry. It reviews various types of polymers, their applications in tissue engineering, and their potential to enhance dental tissue regeneration through biocompatibility, structural support, and integration with bioactive molecules, stem cells, and growth factors. It aims to provide an overview of current advancements and propose future directions for improving scaffold technologies in dental and craniofacial tissue repair.

## 2. Polymers in Regenerative Dentistry

Nowadays, common materials used in dentistry include amalgam, resin, ceramics, glass ionomer, calcium hydroxide, gold, zinc oxide eugenol, etc. [14]. In clinical practice, they are applied as temporary dressings, cements, impression materials, or lining materials [15]. Polymeric materials have also found use in preparing complete or partial dentures. Additionally, they can also be utilized as liners, sealants, or cements. Significant representatives are poly(methyl methacrylate), polyvinyl chloride, or phenol formaldehyde [16]

As mentioned previously in the text, dental tissue is physiologically subjected to various stimuli in the oral cavity. For example, the mastication of food generates so called masticatory force. During routine mastication, its amount measures approximately 70 to 150 newtons. Closer examination of this phenomenon revealed that the condition of dental tissues determines its amount [17]. Effective mastication is also dependable on the mechanical properties of dental tissues. For example, properties such as elasticity, hardness, visco-elasticity, or fracture behavior have been studied in order to better understand the importance of the structure of both soft and hard dental tissues [18,19,20]. From the biochemical point of view, food intake directly influences pH levels in oral cavities. It is known that the acidic environment has a negative effect on enamel and prolonged exposure to pH levels under 5.6 can lead to decay formation [21,22]. Thus, maintaining pH levels around 7 in the mouth protects from the onset of pathological processes and also enables oral microbiota homeostasis [23]. The above-described regularities have to be taken into consideration in the context of regenerative dentistry and scaffold engineering. Basic requirements for scaffolds have been widely described [24]. However, every tissue that aims to be reconstructed has its own specifics. Therefore, biomimetics is also a crucial property in order to achieve the successful restoration of dental tissues. Proper selection of polymeric material with subsequent processing can lead to the formation of an advanced biomimetic scaffold applicable in regenerative dentistry [25].

Natural and synthetic polymers have both been applied in order to restore soft and hard dental tissues. The following text firstly describes the general characteristics of the most common polymers that have been studied in the context of regenerative dentistry. Additionally, concrete applications are included to offer a complex overview of polymeric scaffolds and their potential to restore various dental tissues.

### 2.1. Natural Polymers

Together with stem cells and growth factors, scaffolds represent other significant components applied in the tissue engineering of dental structures. Various studies have pointed out the satisfactory biological properties of natural polymer-based materials, such as biocompatibility, nontoxicity, and biodegradability [26,27]. Moreover, natural polymers also occur in the structure of the native extracellular matrix (ECM) [28]. Hence, scaffolds engineered from these substances offer similar structural, mechanical, and biological properties and can mimic the original ECM structure of concrete tissues in the human body [29]. This feature significantly distinguishes them among the materials applicable in regenerative dentistry. However, the extraction of natural polymers often presents a difficult procedure. Moreover, certain risks of immunogenicity and the rapid degradation rate of these materials present other drawbacks [30]. Fortunately, advanced material research and technologies enable us to overcome these limitations.

Naturally occurring polymers can be further categorized as protein-based and carbohydrate-based biopolymers (Table 1). Both groups have already been studied in the context of scaffold manufacturing [31]. The following text will focus on natural biomaterial scaffolds and their application in regenerative dentistry.

#### 2.1.1. Collagen

Being one of the most distributed proteins, collagen presents an essential structural component of ECM in living organisms [32]. So far, more than 28 types of collagens have been discovered. The most common types in the human body are collagen types I–IV [33]. Collagen plays a crucial mechanical role in connective tissues. This feature is generated from its rigidity and resistance to stretching [34]. Because of its natural occurrence in the human body, scaffolds based on collagen exhibit excellent properties, such as biocompatibility, biodegradability, and mechanical strength. In addition, this polymer is favorable for cells and enhances their attachment, proliferation, or differentiation [35]. Electrospinning enables the creation of fibrous scaffolds with the desired architecture [36]. The importance of collagen scaffold design in dental pulp regeneration was highlighted in a study by Zhang et al. [37]. Electrospinning and subsequent freeze-drying methods fabricated three-dimensional (3D) fibrous collagen matrices. The engineered scaffolds varied in pore sizes. The results showed that all matrices were biocompatible, and larger pores (pore size of 65 μm and 125 μm) enhanced the biological properties of seeded stem cells and thus supported the formation of pulp-like tissue. The collagen scaffold in a hydrogel form was also injected to restore damaged periodontal tissue [38].

#### 2.1.2. Fibrin

Fibrin is a biodegradable polymer, also described as a natural sealant, because it is formed from fibrinogen right after the injury. It also plays a crucial role in the mechanisms of hemostasis, thrombosis, and wound healing [39]. As a biocompatible matrix, it can be applied as a porous scaffold or hydrogel to restore damaged tissues [40]. Fibrin hydrogel scaffolds especially garner attention as they can be easily loaded with bioactive molecules. This could help to better control the rate of in situ tissue regeneration [41]. Its rapid degradation rate presents drawbacks. On the other hand, this characteristic can be altered by conjugating with other polymers [42]. Blood also presents a source of fibrin, as it is the main component of the blood clot matrix [43]. Several studies described favorable characteristics of fibrin-based scaffolds in regenerative endodontics [44,45,46]. The obtained data indicated that hydrogel scaffolds could provide the required conditions to maintain the bioactivity of the stem cells. Moreover, the controlled release of bioactive and antibacterial molecules was also achieved.

#### 2.1.3. Silk

Described as the most challenging natural fiber, silk is a fibrous protein polymer produced by numerous taxonomic families, such as silkworms, glowworms, spiders, etc. [47]. Two specific proteins, fibroin and sericin, make up its structure in a raw state [48]. Because of its good biological properties, silk-based scaffolds have been heavily studied for soft and hard tissue engineering [49]. Silk fibroin (SF) matrices showed great potential due to biocompatibility and biodegradability [50]. Moreover, SF can be processed into various scaffold formats, e.g., hydrogels, sponges, films, nanoparticles, or insoluble implants [51]. In regenerative dentistry, silk-based scaffolds proved potential as these matrices could enhance neo-vascularization and suppress excessive inflammation at the damaged site [52]. Moreover, personalized, shape-memory SF–magnesium scaffolds were developed in a study by Mao et al. [53]. A scaffold was applied to restore irregular bone damage. The results showed that the engineered matrix could enhance bone regeneration, as it promoted the biological activity of bone marrow stem cells under in vitro conditions. These outcomes indicate the noteworthy potential of SF scaffolds to also be applied in regenerative dentistry to restore hard tissues.

#### 2.1.4. Laminin

Laminin is a fibrous glycoprotein component present in the ECM of all animals [54]. The effect on the cells underlines its significant role. Laminin directly impacts cell adhesion, migration, and differentiation [55]. Laminin has been used as a scaffold to repair neural tissue, salivary glands, and dental pulp [56,57,58].

#### 2.1.5. Demineralized Dentin Matrix

Demineralized dentin matrix (DDM) is a natural scaffold derived from extracted human teeth [59]. With superior biological properties, DDM has been applied for bone as well as soft dental tissue regeneration [60]. Dentin consists primarily of the inorganic matrix, and about 30% of its weight is represented by collagenous and non-collagenous proteins together with water [61]. The main advantageous characteristics of DDM are excellent biocompatibility, low immunogenicity, and good mechanical properties. Moreover, its osteoinductive and osteoconductive capacities have also been proven [62]. Because of the origin, released bioactive molecules positively affect dentinogenic events [63]. Several studies have described its regenerative potential to the heal dentin–pulp complex or alveolar bone tissue [64].

#### 2.1.6. Gelatin

Gelatin is a biopolymer obtained by thermal denaturalizing collagen with the gelation ability. It is already heavily used in medical and pharmaceutical industries due to good biocompatibility, biodegradability, and low antigenicity. The United States Food and Drug Administration (FDA) categorizes this biopolymer as Generally Recognized as Safe (GRAS) [65]. Moreover, its low-cost processability enables the formation of scaffolds of various forms, e.g., hydrogels, microspheres, or 3D sponges [66]. In an animal study, Londero et al. applied a gelatin-based scaffold to repair necrotic pulp and apical periodontitis [67]. When combined with blood clots, histological analysis revealed a higher rate of neovascularization and the formation of roots and mineralized tissue. When blended with bioactive glass, a composite gelatin-based scaffold enabled proliferation, migration, and odontogenic differentiation of bone marrow mesenchymal stem cells [68]. In another study, freeze-drying was used to engineer gelatin-based scaffolds loaded with slow-releasing antibiotics [69]. As inflammation based on bacterial infection significantly deteriorates tissue regeneration, this method offers a promising approach to dental tissue restoration. Ribeiro et al. studied a gelatin-based hydrogel in the context of regenerative endodontics [70]. The authors investigated its potential to be applied as a delivery system for the controlled release of chlorhexidine, which is a crucial antiseptic agent. The results showed good cytocompatibility and mechanical as well as structural properties. In addition, a slower degradation rate of chlorhexidine-loaded hydrogel provided the prolonged release of the antiseptic agent.

Microspehere is a specific spherical form of scaffolds that can be loaded with bioactive molecules, thus directly impacting tissue regeneration in situ. Gelatin methacrylate microsperes were studied in order to improve vascularization in endodontic regeneration [71]. In addition, gelatin microsperes can also be loaded with various drugs or cells and directly influence the healing process at the defected site of tissue [72,73]. Gelatin can also be applied in advanced technologies such as 3D printing. Lee et al. engineered a highly elastic 3D gelatin-based composed scaffold intended for hard tissue regeneration [74]. When seeded with mesenchymal stem cells, osteogenic differentiation was successful.

#### 2.1.7. Cellulose

Cellulose is a linear structural polymer consisting of glucose units [75]. It occurs abundantly in nature, specifically in the cell walls of plants. Several types of bacteria are also significant cellulose producers with exceptional mechanical properties, biocompatibility, and biodegradability [76]. When comparing plant and bacterial cellulose, plant cellulose has lower purity, tensile strength, and water content than cellulose obtained from bacteria [77]. Thus, bacterial cellulose has become a noteworthy polymer studied for various medical applications in the form of scaffolds [78]. Additionally, cellulose nanofibers and hydrogels have been the point of interest in numerous experiments [79]. In a study carried out by Jain et al. [80], the bacterial nanocellulose scaffold was tested in the context of its potential application in regenerative dentistry. Cytotoxicity, structure, and physical properties were tested. In addition, the potential of the material to enhance the biological response of the seeded stem cells was also evaluated. The results showed that the manufactured nanocellulose scaffold was non-toxic, and cells could attach and proliferate on its surface. Moreover, seeded stem cells could also undergo odontogenic differentiation.

#### 2.1.8. Alginate

Alginate presents a highly biocompatible and biodegradable polymer harvested from brown algae and certain bacteria [81]. A stable hydrogel is produced in the presence of divalent ions [82]. The outstanding characteristic of the alginate hydrogel is its structural resemblance with ECM [83]. Thanks to this mimicry, the potential of alginate hydrogel applications in regenerative medicine is very high. Moreover, mechanical properties can be easily tuned to provide the right environment for encapsulated and adjacent cells [84]. Several studies described loaded alginate-based hydrogels with growth factors or stem cells as promising dentin–pulp regeneration tools [85]. An experiment conducted by Ferjaoui et al. [86] pointed out the importance of the hydrogel’s mechanical properties in obtaining a satisfactory cellular response, especially osteogenic differentiation. The results proved a superior biological response of loaded dentin pulp stem cells in the alginate-based hydrogel with high stiffness.

#### 2.1.9. Hyaluronic Acid

Hyaluronic acid (HA) is a non-sulfated glycosaminoglycan consisting of repeating disaccharide units [87]. HA presents an essential component of ECM, mainly in connective tissue and synovial fluid. It also occurs in human dental pulp, maintaining its morphology [88]. In addition, HA also influences the proper formation of dentin and enamel during odontogenesis [89]. In regenerative dentistry, HA has been applied as a scaffold in the form of a hydrogel and sponge [90]. Studies highlighted its potential to restore soft and hard dental tissues [91]. When loaded with stem cells, HA-based scaffolds provided favorable conditions for their proliferation, migration, and differentiation [92]. Unfortunately, worse mechanical properties and the rapid degradation of HA hydrogels need to be considered. However, these drawbacks can be overcome by chemical and structural modifications [93].

#### 2.1.10. Chitosan

Chitosan is a polysaccharide-based polymer derived from chitin, which is present in the exoskeleton of crustaceans. It is a biocompatible, biodegradable, eco-friendly material with good biomechanical properties [94]. The main characteristics of chitosan include antibacterial, mucoadhesive, hemostatic, and analgesic properties [95]. When used as a material for scaffold engineering, chitosan impacts the formation of porous architecture [96]. When blended with gelatin, the chitosan-based scaffold obtained better biological, structural, and antibacterial properties, which were studied in regenerative endodontics [97,98].

### 2.2. Synthetic Polymers

Synthetic polymers (Table 2) are widely utilized in regenerative dentistry due to their tunable properties, biocompatibility, and ease of fabrication. These materials are typically used as scaffolds to support tissue regeneration, offering a controlled structure for cellular attachment and growth [99]. Key characteristics include their customizable mechanical strength, biodegradability, and ability to deliver bioactive molecules [100]. Common examples, such as polylactic acid (PLA) and polycaprolactone (PCL), can be tailored to mimic the extracellular matrix, facilitating bone and periodontal tissue repair [101,102]. Despite their advantages, challenges remain in achieving optimal integration and matching the mechanical properties of native dental tissues. Additionally, their degradation products might be toxic. Therefore, a precise analysis of cytotoxicity should provide an early-stage evaluation under in vitro conditions [26].

#### 2.2.1. Polylactic Acid

PLA is a popular synthetic polymer in tissue engineering, recognized for its biocompatibility, controlled biodegradability, and mechanical durability. This material is produced from renewable resources such as carbon dioxide, wheat, corn, and rice. PLA’s degradation products are non-toxic to humans and were approved by the FDA for direct contact with biological fluids [103]. Moreover, PLA represents a three-dimensionally printable polymer, which quickly found its application in human medicine, including regenerative dentistry. It is most used in the regeneration of hard tissues, mainly in combination with hydroxyapatite. For instance, Russias et al. [104] fabricated an osteoinductive PLA/hydroxyapatite composite scaffold with mechanical properties that match those of bone. More recently, Khosronejad et al. [105] prepared a PLA/hydroxyapatite/gelatin/hesperidin scaffold colonized by stem cells, positively affecting bone regeneration in rats with mandibular defects. In a different study, PLA was also used as part of a triple-layered barrier membrane to prevent the unwanted process of mineralization on the membrane’s outer surface in oral environments [106]. 

#### 2.2.2. Polyglycolic Acid

Polyglycolic acid (PGA) is a lactic and glycolic acid copolymer with tunable biodegradation. It is also characterized by great mechanical strength [107]. Another advantageous property is porosity, which affects diffusion, neovascularization, and cellular attachment [108]. PGA is often combined with other materials, such as polymers and ceramics, to improve its properties. It is commonly used to create porous scaffolds with precisely controlled pore sizes and mechanical characteristics. These scaffolds provide a temporary framework for bone cells to attach, proliferate, and generate new bone tissue [109]. PGA is, therefore, ideal for guided bone regeneration as it promotes new bone formation [110].

#### 2.2.3. Polycaprolactone

Polycaprolactone (PCL) is a semi-crystalline aliphatic polyester. It is a widely acknowledged synthetic FDA-approved polymer known for its biocompatibility, biodegradability, ease of fabrication, and mechanical strength, making it ideal for bone tissue engineering [111]. PCL has moderate tensile strength, typically ranging from 10 to 50 MPa, depending on its molecular weight and processing method. PCL maintains mechanical integrity under physiological conditions, which is critical for applications like tissue scaffolding or load-bearing implants. Moreover, PCL can incorporate bioactive molecules, growth factors, mesenchymal stem cells, and minerals (like hydroxyapatite), enhancing bone regeneration [112]. Moreover, PCL scaffolds or membranes support the regeneration of periodontal tissues, including cementum and periodontal ligament [113].

#### 2.2.4. Polyethylene Glycol

Polyethylene glycol (PEG) can be chemically modified and crosslinked, forming hydrogels with tunable mechanical properties and degradation rates. This versatility allows for creating PEG-based scaffolds that closely mimic the native extracellular matrix (ECM) environment, facilitating cell adhesion and proliferation in various tissue engineering approaches. PEG has emerged as a highly versatile synthetic polymer extensively employed in regenerative medicine applications due to its biocompatibility, hydrophilicity, and low immunogenicity [114]. The FDA has approved it for various clinical uses [115]. PEG can form hydrogels with tunable mechanical properties and degradability, making them suitable for tissue engineering scaffolds [116,117]. These hydrogels can mimic the extracellular matrix and support cell growth and differentiation [118]. PEG hydrogels can encapsulate cells, providing a 3D environment for tissue regeneration. For example, bone marrow stromal cells encapsulated in PEG hydrogels showed higher viability than other materials [117]. PEG can be modified with bioactive molecules to enhance cell adhesion, proliferation, and differentiation [119]. This allows for creating ECM-mimetic scaffolds that can guide specific cellular responses. PEG-based systems are used for controlled drug delivery, including biomolecules and low molecular weight drugs [118]. PEG hydrogels have been used in various tissue engineering applications, including bone, cartilage, and nerve regeneration [118,120,121,122,123,124]. While PEG itself is not biodegradable, it can be combined with biodegradable polymers to create materials with controlled degradation rates [125].

#### 2.2.5. Poly(Propylene Fumarate)

As an implantable material in regenerative dentistry, poly(propylene fumarate) (PPF) is a promising biomaterial for polymer-based scaffolds. Its key attributes and applications based on the research include biodegradability, mechanical properties, biocompatibility, osteoconductivity, controlled degradation, and cell support. The biodegradability of PPF is well researched. PPF is a biodegradable polyester that degrades into propylene glycol and fumaric acid, which can be cleared from the body through normal metabolic processes [126]. Concerning its mechanical properties, PPF-based scaffolds exhibit properties similar to trabecular bone, with compressive strengths ranging from 109 ± 2 to 133 ± 6 MPa and compressive moduli of 146 ± 11 to 161 ± 27 MPa [127]. Studies have shown that PPF scaffolds are biocompatible and do not induce foreign body or inflammatory responses [128]. Regarding osteoconductivity, PPF can be combined with materials like β-tricalcium phosphate (β-TCP) to improve this property [128]. PPF can also be 3D printed using stereolithography, allowing for the creation of patient-specific implants. The degradation rate of PPF can be tuned by adjusting its molecular weight, making it suitable for various dental applications [129]. PPF scaffolds can support human dental pulp stem cells’ adhesion, proliferation, and differentiation (DPSCs) [130]. These properties make PPF a versatile material for various applications in regenerative dentistry, including bone regeneration, guided tissue regeneration, and as a carrier for growth factors or stem cells.

#### 2.2.6. Polyurethane

Polyurethanes (PUs) represent a broad class of polymers known for their mechanical robustness, biocompatibility, flexibility, and tunable properties, rendering them suitable for various biomedical applications, including dental tissue engineering [131,132]. Their highly versatile chemical structure allows for incorporating various soft and hard segments, enabling the adjustment of stiffness, elasticity, and biodegradability. This tunability makes PU-based scaffolds particularly attractive for dental applications, where dynamic loading conditions and cyclic stresses are frequently encountered. PU is a promising polymer-based scaffold material for implantable applications in regenerative dentistry [133,134]. The following are key aspects of PU scaffolds in the context of biocompatibility, mechanical properties, porosity, biodegradability, versatility, fabrication methods, and cell support. PU scaffolds demonstrate good biocompatibility, supporting cell adhesion, proliferation, and differentiation of dental-derived cells [135,136]. PU scaffolds can be tailored to match the mechanical properties of dental tissues, with compressive strengths ranging from 12.9 to 116.7 kPa. PU scaffolds can achieve high porosity (80–87%), which is crucial for cell infiltration and tissue ingrowth [132]. PU scaffolds can be designed to degrade at controlled rates, allowing for gradual replacement by regenerated tissue [137]. PUs can be combined with bioactive materials like hydroxyapatite to enhance osteoconductivity and osteoinductivity [137,138]. PU scaffolds can be produced using various techniques, including 3D printing and electrospinning, allowing customized designs [139,140]. PU-based scaffolds present versatile and promising materials in regenerative dentistry, particularly for bone and dental pulp regeneration applications.

#### 2.2.7. Polyhydroxyalkanoates

Polyhydroxyalkanoates (PHAs) are a group of biodegradable polymers produced by certain microorganisms as intracellular carbon and energy storage compounds. PHAs are a promising alternative to petroleum-based plastics due to their biodegradable nature, renewability, and non-toxic properties [141]. HAs are biodegradable in both aerobic and anaerobic environments. This makes them environmentally friendly and an attractive alternative to synthetic plastics, which can persist in the environment for centuries [142]. A great advantage of PHAs is the ability to modify their mechanical properties by changing the monomer composition. For instance, innovative PHA blends with optimized monomer ratios can now be engineered to achieve a wide range of desired characteristics, such as enhanced flexibility, tensile strength (ranges from 20 to 40 MPa), and durability [143,144,145,146]. PHAs can serve as scaffolds for dental tissue regeneration, such as alveolar bone, periodontal ligament, and dentin [147]. They can be used as coatings on dental implants and biodegradable wound dressing for intraoral surgical procedures to improve their biocompatibility, promote healing, and reduce the risk of infection [148].

## 3. Clinical Application of Polymeric Scaffolds in Regenerative Dentistry

In the context of clinical application, polymeric scaffolds were tested in order to restore endodontium, periodontal tissues, and craniofacial tissues including nerves and salivary glands [16,149]. Depending on the study design, natural as well as synthetic polymer-based scaffolds were evaluated. The following text offers examples of clinical experiments applying concrete polymers in order to evaluate their regenerative potential.

### 3.1. Regenerative Endodontics

Regenerative endodontics aims to recover the pulp–dentin complex. This complex is structurally and functionally united [150]. Hence, pathological conditions influencing endodontium often impact both pulp and dentin and cause severe pain. Moreover, if untreated, periodontal tissues can be affected as well [151]. Currently, a clinically approved approach tending to repair immature permanent teeth with necrotic pulp is based on pulp revascularization, in which blood clot acts as a scaffold [152]. In several studies, a collagen sponge was placed over the blood clot to fix or to be used as a capping material [153,154]. Jiang et al. conducted a randomized clinical trial in which the efficacy of root development with the application of a collagen membrane was evaluated [155]. The results showed that these membranes acting as intracanal scaffolds promoted the deposition of dentin in the middle thirds of the roots. When compared with the approach using only blood clot, collagen membranes proved better overall clinical results. However, other parameters which were also compared (root length, dentin thickness in apical area, disclorotation, i.a.) did not show significant differences between the control and experimental groups. The potential of collagen-based composite scaffolds with synthetic carbonated apatite (SynOss Putty, Collagen Matrix, Oakland, NJ, USA) was also clinically evaluated [156]. SynOss Putty was applied as a scaffold for regenerative endodontic treatment applied in immature noninfected human teeth. The outcomes revealed that the best histological outcomes in the context of root development were obtained when applied together with blood clot, as hard tissue formation in teeth was detected. In addition, SynOss Putty also proved its potential for the treatment of apical periodontitis [157]. Nakashima et al. utilized a collagen scaffold as a carrier for autologous mobilized dental pulp stem cells together with granulocyte colony-stimulating factor (G-CSF) [158]. The aim of this pivotal study was to evaluate the safety and efficacy of autologous cell transplantation into pulpectomized teeth. Not only were no post-transplantation adverse effects detected, but the successful formation of the dentin–pulp complex was achieved. A collagen scaffold obtained good results in the pulp restoration of mature necrotic teeth as well [159].

Chitosan-based scaffolds were also clinically evaluated in the context of regenerative endodontics [160]. Alshahhoud et al. compared the regenerative potential of three different scaffolds: blood clot, native chitosan with blood clot, and an enzymatically modified chitosan and blood clot scaffold. The aim was to repair periapical lesions and to evaluate tooth sensibility. A total of 24 participants were involved. The best results were observed 6 months after the procedure in the group that received the combined scaffolds of blood clot and enzymatically modified chitosan.

### 3.2. Periodontal Tissue Regeneration

Periodontal tissue includes alveolar bone, periodontal ligament, and cementum. These structures are often affected by periodontitis and their regeneration is based on guided tissue regeneration [149]. Specific barrier membranes are applied in order to regulate the healing process [161]. Non-resorbable as well as resorbable barriers had been heavily applied and compared [162]. According to the latest clinical studies (publishing years 2020–2025), collagen was mainly utilized as a resorbable material for guided bone regeneration. However, the results of the studies did not specifically highlight the superiority of these scaffolds over non-resorbable membranes [163,164,165].

A scaffold derived from synthetic polymer polycaprolactone (PCL) was evaluated in a clinical study in the context of hard tissue regeneration [111]. A total of 10 patients with a defected maxillary or mandibular bone were included. Clinical parameters such as swelling, infection, and graft exposure were observed at various time intervals. In addition, specific radiographs were used to evaluate the condition of the defected bone. The results showed that PCL scaffolds could not significantly repair bone defects as they had the tendency for dehiscence.

Periodontal soft tissue regeneration is also a challenging field within regenerative dentistry. Yakout et al. applied hyaluronic gel in order to repair gingival tissue after gingivectomy [166]. The outcomes revealed that a 2% hyaluronic acid gel had a significant effect on gingival wound healing. ECM-derived scaffolds also represent favorable materials. Decellularized human dermis, human amniotic membrane, and various forms of collagen scaffolds have been applied to restore gingival recessions or to provide augmentation at dental implant sites. The results confirmed the potential of ECM-derived scaffolds to be applied clinically in regenerative dentistry [167,168,169].

### 3.3. Hard Tissue Regeneration

The regeneration of dental hard tissues remains challenging as they are tightly connected with other structures such as nerves, blood vessels, ligaments, cartilages, muscles, and also teeth [170]. The temporomandibular joint is a frequent site of pathology which can be affected by autoimmune disorders, osteoarthritis, or disk displacements [171]. Despite the fact that various natural and synthetic polymers have been studied under in vitro and in vivo conditions, clinical outcomes are still lacking [172]. Collagen-based membranes are favorable materials for bone tissue regeneration as they have a direct impact on osteogenesis and the regeneration of periodontal tissues. In addition, they degrade enzymatically, so no secondary intervention for their retrieval is needed [165]. In a clinical study conducted by Saputra et al., collagen-based membranes derived from pericardium and amnion membrane were applied in order to repair a large alveolar bone defect caused by radicular cysts [173]. Although promising results were obtained, due to the small number of participants (two women), statistical significance could not be determined. In another study, advanced technologies (3D printing) were applied to engineer patient-tailored 3D scaffold based on PCL. Staged alveolar bone augmentation was performed on a single patient and parameters such as bone gain and implant stability were evaluated [174]. The results showed that the scaffold fulfilled osteoconductive as well as osteoinductive roles. Allogenic bone materials are commonly used graft materials for bone reconstruction. In a study carried out by Kovac et al., they were combined with collagen membranes in order to repair horizontal bone defects in pediatric patients [175]. Additionally, an autologous graft together with a xenogenous matrix were used as well and this approach could successfully restore a horizontal ridge defect.

## 4. Future Directions

Recently, regenerative dentistry has undergone transformative advancements driven by integrating innovative (smart) biomaterials and tissue engineering approaches. Current research emphasizes the development of scaffolds derived from natural and synthetic polymers, which provide critical support for cellular proliferation, differentiation, and tissue regeneration [5,176].

Natural polymers such as collagen, chitosan, and alginate offer excellent biocompatibility and mimic the extracellular matrix (ECM), facilitating cell attachment and proliferation. Incorporating bioactive molecules into these scaffolds enhances their regenerative potential, particularly in soft and hard tissue engineering [28,31]. Synthetic polymers stand out for their tunable mechanical properties and degradation rates. These materials are increasingly combined with nanotechnology to develop multifunctional scaffolds catering to patient-specific requirements [99,177].

Searching for suitable combinations of natural and synthetic biomaterials enables the creation of hybrid scaffolds that leverage both strengths, such as improved mechanical strength and biological compatibility [178]. Future research will also focus on incorporating stimuli-responsive biomaterials capable of releasing bioactive agents in response to environmental cues, representing a promising direction. These “smart” scaffolds could significantly enhance the precision of regenerative therapies and enable personalized solutions [100].

Advances in 3D and 4D bioprinting are also expected to revolutionize scaffold design, allowing for the fabrication of highly customized, patient-specific implants replicating complex dental and craniofacial structures [179]. Importantly, 3D printing was also utilized in clinical practice, which enabled the application of patient-specific scaffolds. Vast research is still going on under in vitro conditions, which are frequently oriented on bone tissue engineering [180]. Smart 3D-printed scaffolds offer precise architecture and can be loaded with various antimicrobial factors and regulatory molecules, which can enhance cellular response, neo-vascularization, or the formation of neural tissue in situ [181,182,183]. Moreover, 4D printing also draws attention, as it allows for the creation of scaffolds that can adjust their morphology and functionality due to received stimuli [184]. This approach holds great potential for the successful integration of the scaffold in situ, as the matrix should alter its properties in accordance with inner in vivo conditions. This could also help to prevent the rejection of the transplant, which presents a significant drawback of transplanted tissue-engineered constructs [185]. For 4D printing, novel bioinks were also tested. They have to fulfill several requirements. Most importantly, they have to be responsive to physical or chemical changes, such as mechanical load, magnetic field, heat, light, moisture, or pH. Multiple polymers have been applied in 4D printing, mostly as composite materials, e.g., PCL, PLA, PUs, and PPF. Natural polymers were also applied in hydrogel forms, e.g., collagen, agarose, cellulose, alginate, chitin, fibrin, and gelatin [186].

Biomaterials research should aim to integrate knowledge from stem cell research about the possibilities of their acquisition, preconditioning, and large-scale production in a closed system for safe use in colonizing biomaterials to mimic tissues [187]. The research will also focus on the role of extracellular vesicles (EVs) and paracrine signaling mechanisms. EVs play a crucial role in cell-to-cell communication by transporting diverse bioactive molecules, such as proteins, lipids, and nucleic acids. Their natural ability to influence cellular behavior and the microenvironment makes EVs highly promising in regenerative dentistry and biomaterial engineering [188]. Integrating EVs into biomaterials offers an innovative approach to enhancing the efficacy of regenerative therapies. Biomaterials can be designed to act as delivery platforms for EVs, ensuring the controlled and sustained release of their therapeutic contents [189]. For example, hydrogels and scaffolds can be engineered to encapsulate EVs and release them in response to specific environmental triggers such as pH changes, enzymatic activity, or temperature shifts. This targeted delivery system can improve the precision and effectiveness of treatments, reducing the risks of off-target effects [190]. In the context of regenerative dentistry, exosomes derived mostly from dental pulp stem cells were applied to repair the pulp–dentin complex and bone [191,192]. Additionally, they were also utilized as bioactive agents promoting neo-vascularization and nerve regeneration [193,194].

Future research must address biosafety issues, especially malignant cell transformation, because it enhances the credibility of biomaterials and regenerative medicine applications [195,196,197]. It paves the way for safer, more effective therapies that improve patient outcomes while minimizing risks. Such efforts also foster trust among regulatory bodies, healthcare providers, and patients, accelerating the translation of innovations from bench to bedside [198].

Lastly, further research must also address environmental and cost considerations by focusing on sustainable and scalable production methods for biomaterials [199].

## 5. Discussion

The present review demonstrates that polymer-based scaffolds represent a highly promising avenue for advancing regenerative dentistry. In particular, the body of evidence indicates that both natural and synthetic polymers contribute distinct advantages to scaffold design, while also presenting unique challenges that must be overcome for successful clinical translation.

Natural polymers such as collagen, fibrin, silk, and chitosan have attracted considerable attention due to their excellent biocompatibility, intrinsic bioactivity, and ability to closely mimic the native extracellular matrix (ECM) [26,27,29]. Collagen-based scaffolds, for example, support cellular attachment and differentiation and have shown significant promise in dental pulp regeneration [32,33,34,35,36,37,38]. However, these materials often suffer from rapid degradation and potential immunogenicity, which can compromise the long-term mechanical stability required in load-bearing applications [30]. Similarly, while fibrin and chitosan offer bioactive environments that favor cell proliferation and tissue repair, their limited mechanical strength necessitates further modification or reinforcement [39,40,41,42,43,44,45,46,94,95,96,97,98].

In contrast, synthetic polymers such as polylactic acid (PLA), polycaprolactone (PCL), and polyethylene glycol (PEG) offer highly tunable mechanical properties, controlled degradation rates, and ease of processing [103,104,105,106,111,112,113,114,115,116,117,118,119,120,121,122,123,124,125]. These characteristics enable the fabrication of scaffolds that can be precisely engineered to meet the biomechanical demands of dental tissues. Nevertheless, the inherent bioinertness of many synthetic polymers limits cell adhesion and proliferation unless they are modified with bioactive molecules or blended with natural polymers [26,99]. Consequently, hybrid scaffold systems that integrate the biocompatibility of natural materials with the mechanical resilience of synthetic ones have emerged as a particularly attractive strategy [178].

The clinical applications discussed herein underscore both the potential and the current limitations of polymer-based scaffolds. In regenerative endodontics and periodontal tissue regeneration, early clinical studies have demonstrated that scaffolds—whether derived from natural or synthetic sources—can facilitate tissue repair and promote the regeneration of complex dental structures [111,155,158]. Yet, these results are often variable and, in some cases, statistically underpowered due to small sample sizes or limited follow-up periods. Moreover, the challenges of achieving adequate vascularization, immune compatibility, and long-term integration with host tissues persist [111,159,169].

Advancements in artificial intelligence allowed simplified auto-segmentation from CBCT (Cone Beam Computed Tomography) data in dental environments, changing the traditional roles of personnel. Similarly, improvements in manufacturing technologies, including 3D printing, and now emerging 4D bioprinting, are set to further refine scaffold architecture and enable the production of patient-specific implants with spatially controlled properties [179,181,182,183,184]. The integration of nanotechnology and stimuli-responsive materials is expected to offer dynamic platforms that can modulate the release of growth factors or other bioactive agents in response to environmental cues, thereby enhancing the regenerative process [100,177]. Additionally, recent investigations into extracellular vesicles (EVs) and exosome incorporation into scaffold designs highlight a novel approach to boost cell–cell communication and tissue repair [188,189,190,191,192,193,194].

In summary, while significant progress has been made in elucidating the properties and applications of polymer-based scaffolds in regenerative dentistry, several critical challenges remain. Future research should focus on optimizing the balance between biodegradability and mechanical integrity, enhancing the bioactivity of synthetic platforms, and ensuring the scalability and reproducibility of hybrid scaffold systems. Addressing these issues will be pivotal for advancing these technologies from preclinical studies to reliable, effective clinical therapies that can meet the complex demands of dental tissue regeneration.

## 6. Conclusions

In conclusion, applications of polymer-based scaffolds within regenerative dentistry can more effectively address clinical challenges by bridging interdisciplinary innovations, paving the way for minimally invasive, patient-centered solutions. Researchers and clinicians must focus on translating these advancements into clinical practice to improve patient outcomes and redefine the standards of care in dentistry.

## Figures and Tables

**Table 1:** Overview of natural polymers studied in context of regenerative dentistry.

Polymer-Based Scaffold	Advantages	Disadvantages	References
Collagen	biocompatibility biodegradability support cell attachment osteoinductive properties good tensile strength various forms of scaffolds	↓ post-implantation stability scaffold shrinkage rapid degradation possible immunogenicity	[32,33,34,35,36,37,38]
Fibrin	biocompatibility controlled biodegradability bioactive surface elasticity viscous properties	↓ mechanical properties scaffold shrinkage possible immunogenicity	[39,40,41,42,43,44,45,46]
Silk	biocompatibility mechanical properties toughness various forms of scaffolds	its processing presents ecological burden long degradation rate	[47,48,49,50,51,52,53]
Laminin	enhances cellular behavior regeneration of soft tissue	possible immunogenicity	[54,55,56,57,58]
Demineralized dentin matrix	mechanical properties	process of demineralization	
	contains bioactive molecules osteoinduction osteconduction		[59,60,61,62,63,64]
Gelatin	biocompatibility	rapid enzymatic degradation	
	biodegradability low antigenicity low-cost processing various forms of scaffolds	↓ mechanical properties	[65,66,67,68,69,70,71,72,73,74]
Cellulose	enhances cell attachment provides 3D architecture	↓ biodegradability	[75,76,77,78,79,80]
	adjustable properties		
Alginate	biocompatibility	rapid degradation	
	biodegradability supports cellular behavior form of hydrogel	↓ mechanical strength	[81,82,83,84,85,86]
Hyaluronic acid	biocompatibility bioactivity enhances cellular behavior nonthrombogenic	rapid degradation ↓ mechanical strength	
			[87,88,89,90,91,92,93]
Chitosan	biocompatibility biodegradability enhances cellular behavior porous structure antibacterial properties	↓ mechanical strength	[94,95,96,97,98]
		inconsistent behavior	

Arrow means lower.

**Table 2:** Overview of synthetic polymers studied in context of regenerative dentistry.

Polymer-Based Scaffold	Advantages	Disadvantages	References
Polylactic acid	biocompatibility	↓ hydrophilicity	
	regulated biodegradation	brittleness	[103,104,105,106]
	mechanical properties eco-friendly 3D printing application induction of osteogenesis FDA approved	↓ thermoresistance ↓ cell affinity	
Polyglycolic acid	biocompatibility	potential inflammatory response rapid degradation	
	adjustable biodegradation mechanical strength bioactivity porosity can be blended promotes bone regeneration		[107,108,109,110]
Polycaprolactone	biocompatibility	hydrophobic surface	
	biodegradability	toxic solvents	[111,112,113]
	mechanical properties easy processing		
	modification with biomolecules promotes bone regeneration periodontal tissue regeneration FDA approved		
Polethylene glycol	biocompatibility,	poor mechanical strength	
	↓ immunogenicity flexibility mimics ECM can form hydrogels modification with bioactive agents can be blended FDA approved	↓ cell attachment	[114,115,116,117,118,119,120,121,122,123,124,125]
Poly(propylene fumarate)	biocompatibility	difficult handling	
	adjustable biodegradation	hydrophobic surface	[126,127,128,129,130]
	mechanical properties osteoconductivity applicable in 3D printing good cellular response		
Polyurethanes	biocompatibility	potentially toxic degradation products limited use for long-term application	
	mechanical strength flexibility support cellular response injectability porosity blends with superior properties		[131,132,133,134,135,136,137,138,139,140]
Polyhydroxyalkanoates	biocompatibility	crystallinity	
	biodegradation	brittleness	[141,142,143,144,145,146,147,148]
	mechanical properties	long degradation	
	surface modification structural diversity thermal properties bio-origin non-cancerogenic		

Arrows mean lower.

## Data Availability

No new data were created or analyzed in this study. Data sharing is not applicable to this article.
